# Kidney re-transplantation in a child across the barrier of persisting angiotensin II type I receptor antibodies

**DOI:** 10.1007/s00467-020-04879-8

**Published:** 2020-12-23

**Authors:** Annika Gold, Alexander Fichtner, Daniela Choukair, Claus Peter Schmitt, Caner Süsal, Duska Dragun, Burkhard Tönshoff

**Affiliations:** 1 Department of Pediatrics I, University Children’s Hospital Heidelberg, Im Neuenheimer Feld 430, 69120 Heidelberg, Germany; 2 Institute of Immunology, University Hospital Heidelberg, Heidelberg, Germany; 3 Clinic for Nephrology and Critical Care Medicine, Charité-Universitätsmedizin Berlin, Berlin, Germany; 4 Berlin Institute of Health, Berlin, Germany

**Keywords:** Kidney transplantation, Antibody-mediated rejection, Angiotensin type 1 receptor antibodies, Donor-specific HLA antibodies

## Abstract

**Background:**

Approximately 20% of antibody-mediated rejection (ABMR) episodes in the absence of donor-specific antibodies against human leucocyte antigens (HLA-DSA) in pediatric and adult kidney transplant recipients are associated with, and presumably caused by, antibodies against the angiotensin type 1 receptor (AT_1_R-Ab). While the role of AT_1_R-Ab for ABMR and graft failure is increasingly recognized, there is little information available on the management of these patients for re-transplantation over the barrier of persisting AT_1_R-Ab.

**Case:**

We report on a male patient with kidney failure in infancy due to obstructive uropathy who had lost his first kidney transplant due to AT_1_R-Ab-mediated chronic ABMR. Because this antibody persisted during 4 years of hemodialysis, for the 2nd kidney transplantation (living-related transplantation from his mother), he underwent a desensitization regimen consisting of 15 plasmapheresis sessions, infusions of intravenous immunoglobulin G and thymoglobulin, as well as pharmacological blockade of the Angiotensin II (AT II) pathway by candesartan. This intense desensitization regimen transiently decreased elevated AT_1_R-Ab titers, resulting in stable short-term kidney allograft function. The subsequent clinical course, however, was complicated by acute cellular rejection and chronic ABMR due to persistent AT_1_R-Ab and de novo HLA-DSA, which shortened allograft survival to a period of only 4 years.

**Conclusion:**

This case highlights the difficulty of persistently decreasing elevated AT_1_R-Ab titers by a desensitization regimen for re-transplantation and the detrimental effect of the interplay between AT_1_R-Ab and HLA-DSA on kidney transplant survival.

## Introduction

Antibody-mediated rejection (ABMR) plays a significant role in graft loss in both adult [1–3] and pediatric kidney transplant recipients [2, 4]. The majority of these rejections are caused by pre-formed and/or de novo donor-specific antibodies against human leucocyte antigens (HLA-DSA). However, there is a significant subset of patients with histological features of ABMR in the graft biopsy, in whom HLA-DSA cannot be detected in the circulation [5, 6]. In recent years, therefore, there have been increasing efforts directed towards the detection and biological characterization of antibodies against other endothelial targets beside HLA. In the year 2005, the discovery of antibodies against the angiotensin type 1 receptor (AT_1_R) in patients undergoing ABMR without detectable HLA-DSA by Dragun et al. significantly increased our understanding of the role of non-HLA antibodies in the pathophysiology of ABMR [7]. Approximately 20% of ABMR episodes in the absence of HLA-DSA in pediatric and adult kidney transplant recipients are associated with, and presumably caused by, AT_1_R antibodies (AT_1_R-Ab) [8, 9].

AT_1_R-Ab are a group of receptor-activating antibodies (agonists) inducing downstream events such as vasoconstriction, remodeling of the extracellular matrix, and induction of proinflammatory cascades [10]. It has been hypothesized that ischaemia-reperfusion injury increases the expression of donor AT_1_R on vascular smooth-muscle and endothelial cells, predisposing the graft to injury by pre-existing AT_1_R-Ab [11]. Furthermore, AT_1_R-Ab upregulate HLA class II antigens on endothelial cells, thereby potentially enhancing the detrimental effects of HLA-DSA [6]. A few case reports have highlighted the broad spectrum of different clinical phenotypes of AT_1_R-Ab-mediated tissue injury [12–15].

While the role of AT_1_R-Ab for ABMR and graft failure is increasingly recognized, there is little information available on the management of these patients for re-transplantation over the barrier of persisting AT_1_R-Ab. We therefore report here the desensitization for re-transplantation and long-term follow-up of a pediatric patient, who had lost his first kidney allograft due to AT_1_R-Ab-mediated ABMR.

## Case report

We report on a male patient with chronic kidney disease stage 5 in infancy due to obstructive uropathy. He received a first kidney transplant at the age of 3.2 years from a deceased female donor (42 years of age, one HLA-A and one HLA-DR mismatch) in the year 2000.

The initial immunosuppressive therapy consisted of cyclosporin A microemulsion (CsA), mycophenolate mofetil (MMF), and methylprednisolone. On day 7 post-transplant, during a period of inadequate CsA exposure, he experienced acute T cell–mediated rejection with mild to moderate intimal arteritis (BANFF ‘97 Grade IIa), which was treated with methylprednisolone pulses, OKT3, and switch of CsA to tacrolimus. Thereafter, graft function was stable for 6 years post-transplant with a serum creatinine concentration of approximately 1.4 mg/dL. He then experienced a progressive decline of graft function (increase of serum creatinine to 2.5 mg/dL) accompanied by severe arterial hypertension. Kidney allograft biopsy revealed chronic transplant glomerulopathy with partial glomerular sclerosis, interstitial fibrosis and tubular atrophy (IFTA), mild tubulitis, and arterial intimal fibrosis of new onset; C4d staining by immunohistochemistry was negative. These histopathological lesions were at the time categorized as acute vascular and interstitial rejection (BANFF IIb). With today’s histopathological classification, these lesions were consistent with chronic active ABMR. There was no serological evidence of HLA-DSA measured by the LABScreen Single Antigen assays (OneLambda, Thermofischer Scientific Canoga Park, CA), but the serum concentration of AT_1_R-Ab was markedly elevated (112 U/L, reference range <10 U/L). AT_1_R-Ab were initially measured with a bioassay [7], subsequently with a cell-based enzyme-linked immunosorbent assay (ELISA) (CellTrend GmbH, Luckenwalde, Germany, now OneLambda, Thermofischer Scientific Canoga Park, CA). The categories of binding reported in the ELISA test usually indicate negative binding at < 10 U/mL and lower, intermediate binding at 10–17 U/mL, and strong binding at > 17 U/mL [16]. Endothelin type A receptor antibodies (ET_A_R-Ab) were also measured with a sandwich ELISA (CellTrend GmbH, Luckenwalde, Germany, now OneLambda, Thermofischer Scientific Canoga Park, CA). As the potential role of AT_1_R-Ab in refractory vascular rejection had only been published 1 year before the patient’s episode of rapid loss of graft function and onset of arterial hypertension [7], no baseline measurement of non-HLA-antibodies had been performed prior to transplantation.

Despite antirejection therapy with methylprednisolone pulses, increased tacrolimus exposure (target trough level of 10–12 μg/L), and pharmacological blockade of the angiotensin (AT) II pathway by candesartan (0.1 mg/kg body weight per day), graft function rapidly declined. Candesartan has the highest affinity for the AT_1_R and was therefore used [17]. Five therapeutic plasma exchange sessions transiently decreased the AT_1_R-Ab titer from 110 to 25 U/mL, followed by a rapid secondary increase. Transplant function did not recover, and after 6.5 years with a functioning graft, the patient required kidney replacement therapy with hemodialysis. Transplant nephrectomy was performed because of recurrent pyelonephritis and nearly complete loss of transplant function. Histology of the explanted graft showed severe transplant glomerulopathy and severe IFTA. AT_1_R-Ab concentration peaked at 276 U/mL 16 months after transplantectomy, then slowly decreased to 55 U/mL over 2 years but remained persistently elevated (serum concentration around 20 U/mL) in the following years. While being on chronic hemodialysis therapy for 4 years, he experienced recurrent thromboembolic events in his central venous (jugular) catheter and arterio-venous fistula without any other known thrombophilic risk factors except high serum AT_1_R-Ab. Elevated AT_1_R-Ab might have contributed to these recurrent shunt thromboses, as they stimulate coagulation by inducing tissue factor expression and inhibiting fibrinolysis [13]. Antihypertensive medication was slowly weaned; candesartan was stopped after 3 years.

**Fig. 1:**
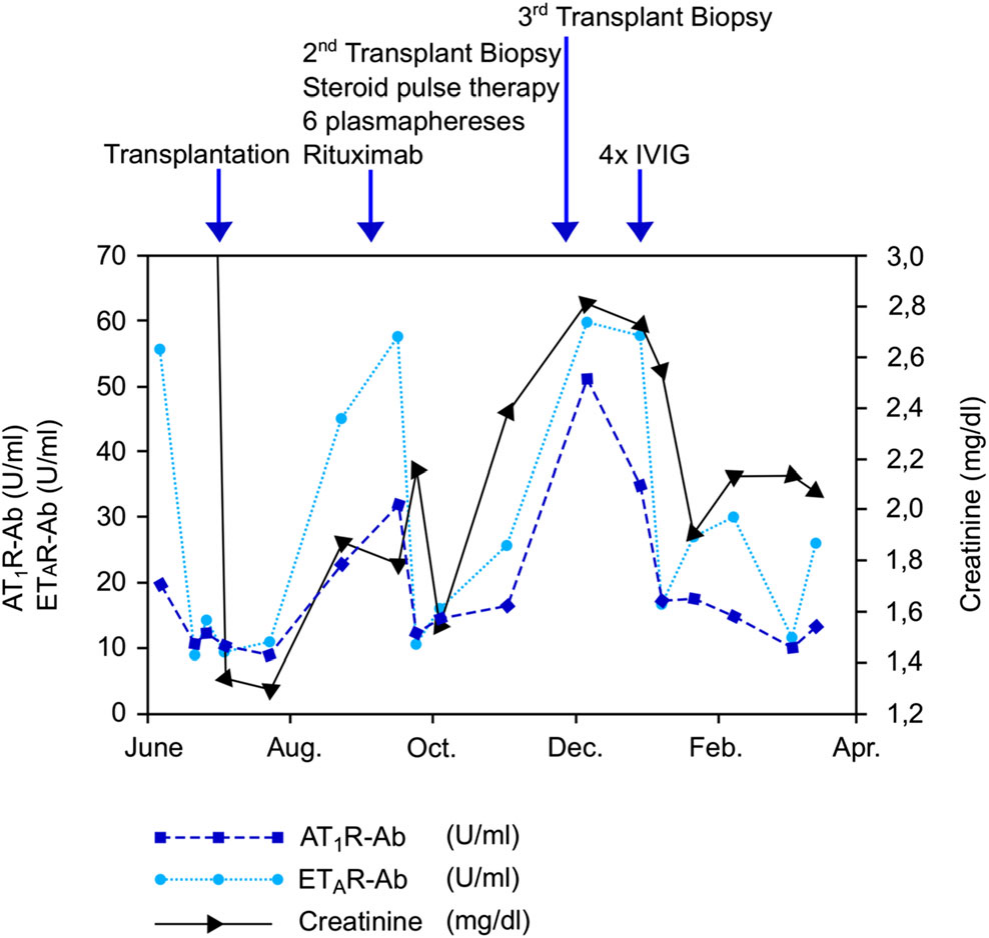
Course of antibodies against the angiotensin type 1 receptor (AT_1_R-Ab), the endothelin type A receptor (ET_A_R-Ab), and graft function (serum creatinine) pre-transplant and during the first 10 months after the second kidney transplantation

At the age of 13 years, he received a 2nd kidney allograft as a living-related transplantation from his mother (one HLA-A, -B, and –DR mismatch each, pre-transplant HLA class I and class II DSA-negative). Because of the persistently elevated AT_1_R-Ab prior to transplantation, he underwent a desensitization regimen consisting of 15 plasmapheresis sessions (three sessions per week over 5 weeks, each time 150% exchange of plasma volume with human albumin 5%), followed by three infusions of intravenous immunoglobulin G (IVIG). The desensitization regimen was based on our local desensitization protocol for kidney transplantation across the HLA and ABO barriers (for adults and children) and the protocol for treatment of rejection episodes due to AT_1_R-Ab in adult patients [7, 18–20], because no published protocol was available for desensitization across the barrier of persisting AT_1_R-Ab. This regimen decreased the AT_1_R-Ab titer from 19.8 to 9.5 U/mL and the ET_A_R antibody titer from 56 to 9.5 U/mL (Fig. 1). For immunosuppressive induction therapy, the patient received 3-times thymoglobulin i.v. (cumulative dose of 4.5 mg/kg body weight) and three sessions of plasmapheresis in the first 10 days post-transplant. Immunosuppressive maintenance therapy consisted of tacrolimus (initial dose 0.3 mg/kg per day), MMF (1200 mg/m^2^ per day), and methylprednisolone. Pharmacological blockade of the AT II pathway by candesartan (0.15 mg/kg body weight per day) was resumed on day 10 post-transplant, and therapy with iloprost (0.5 ng/kg/min infused over 6 hours) for 7 days was initiated to improve renal microcirculation, as iloprost attenuates AT II–mediated vasoconstriction [21]. Initial graft function was excellent. A surveillance allograft biopsy on day 17 post-transplant revealed interstitial borderline rejection without signs of vascular rejection; therefore, tacrolimus exposure was increased (target trough level 10–12 μg/L).

On day 87 post-transplant, he experienced a rapid decline in transplant function, accompanied by an increase in AT_1_R-Ab and ET_A_R-Ab (Fig. 1); in addition, a de novo DSA against the donor mismatch HLA-DQ7 (MFI value > 9000 by the Luminex single antigen assay) was detected. The histopathological evaluation showed acute interstitial rejection (BANFF 1A) with pronounced IFTA. Antirejection therapy consisted of methylprednisolone pulse therapy, six sessions of plasmapheresis, and one dose of rituximab (375 mg/m^2^). Furthermore, the patient again received vasodilatory therapy with iloprost (initially once weekly and thereafter every second week). For-cause kidney allograft biopsies on day 123 and day 168 post-transplant were performed due to decreasing kidney function and rising AT_1_R-Ab and ET_A_R-Ab titers; but there were no histopathological signs of acute rejection or accelerated IFTA. In order to reduce antibody titers, three additional sessions of plasmapheresis were conducted, and anti-humoral therapy with high-dose IVIG (four weekly doses, 1 g/kg body weight per dose) was administered.

In the following 2 years, serum creatinine slowly increased from 2.0 to 2.8 mg/dL. Besides the DSA against donor mismatch HLA-DQ7 (DQB1*03:01), he also developed a DSA against HLA-DQA1 (DQA1*03:03) and transiently against HLA-DR11 (DRB1*11:01) despite adequate immunosuppressive triple therapy with tacrolimus, MMF and steroids, and good treatment adherence. AT_1_R-Ab and ET_A_R-Ab concentrations remained high with a saturation binding at > 40 U/mL at most times. His clinical course was further complicated by recurrent episodes of pyelonephritis and pneumonia, which as inflammatory events might have stimulated HLA-DSA and non-HLA antibody formation. Serum creatinine steadily increased; another allograft biopsy at 4 years post-transplant showed chronic ABMR with mononuclear interstitial infiltration, mild tubulitis, C4d positivity in 25% of the peritubular capillaries, and pronounced IFTA. After 4 years with a functioning graft, he had to resume chronic hemodialysis therapy.

## Discussion

This is the first case report of re-transplantation in a patient who had lost his first graft due to AT_1_R-Ab-mediated chronic ABMR. Our data show that an intense desensitization regimen consisting of plasmapheresis and IVIG (to remove circulating AT_1_R-Ab) and thymoglobulin (to prevent new antibody production) can transiently decrease elevated AT_1_R-Ab titers, resulting in stable short-term kidney allograft function. However, the subsequent clinical course was complicated by acute cellular rejection and chronic ABMR, which shortened allograft survival to a period of only 4 years. It is difficult to assess to what extent the persistently elevated AT_1_R-Ab and ET_A_R-Ab titers contributed to allograft injury of his second graft, because he also developed two de novo HLA-DSA, but the unfavorable course of his first graft in the absence of any HLA-DSA render a pathogenic role of elevated AT_1_R-Ab and ET_A_R-Ab quite likely. It is currently not known whether elevated ET_A_R-Ab have a pathogenic role for graft rejection independent of elevated AT_1_R-Ab. AT_1_R-Ab appear to stimulate the development of de novo HLA-DSA [10], and the negative effect of the interplay between AT_1_R-Ab and HLA-DSA on kidney and liver transplant survival has well been demonstrated [8, 16, 22]. Other case series on patients with only AT_1_R-Ab-mediated graft injury without HLA-DSA report a more favorable outcome with good long-term graft survival and absence of major complications after adequate anti-humoral therapy [7, 15]. Whether the sole presence of pre-transplant AT_1_R-Ab positivity justifies a prophylactic desensitization regimen is still a matter of debate. Carroll et al. investigated in a retrospective single-center study in adult kidney transplant recipients the effect of peri-operative plasma exchange and candesartan in patients with high pre-transplant AT_1_R-Ab positivity and observed that this perioperative regimen may alter the risk of rejection compared to a historical control group [23], but further studies are needed.

An elevated AT_1_R-Ab and ET_A_R-Ab titer may induce severe arterial hypertension. These antibodies lead to a sustained activation of the AT_1_R and ET_A_R, which stimulates vasoconstriction via G-protein coupling [7] and upregulation of the respective receptor expression at the target cell membrane [10]. While we previously observed an association of AT_1_R-Ab positivity and higher systolic blood pressure in pediatric kidney transplanted patients [8], this association was not observed in the study of Pearl et al. [24]. The pathophysiological relevance of these antibodies for arterial hypertension remains therefore to be elucidated. The variable clinical phenotype of high AT_1_R-Ab positivity may be explained by inter-individually different (genotypic) receptor expression at the target cell membrane, different autoantibody epitopes with variable agonistic function, or a desensitization of the post-receptor pathway in endothelial cells due to persisting activation of the AT_1_R [10].

This case also highlights the medical need to develop more effective therapies against elevated AT_1_R-Ab and ET_A_R-Ab. Our conventional multimodal therapeutic approach, which was used in analogy to barrier transplantations in patients highly immunized against HLA antigens, led only to a partial and transient reduction of AT_1_R-Ab and ET_A_R-Ab titers with an overall unsatisfactory clinical course. It remains to be seen whether newer induction regimens with other anti-B cell biologicals such as daratumumab, perhaps in conjunction with the dual AT_1_R and ET_A_R blocker sparsentan, allow a more favorable outcome in these difficult-to-treat patients.
